# The Role of Epigenetic Modification in Tumorigenesis and Progression of Pituitary Adenomas: A Systematic Review of the Literature

**DOI:** 10.1371/journal.pone.0082619

**Published:** 2013-12-18

**Authors:** Matthew Pease, Chao Ling, William J. Mack, Kai Wang, Gabriel Zada

**Affiliations:** 1 Department of Neurosurgery, Keck School of Medicine, University of Southern California, Los Angeles, California, United States of America; 2 Zilkha Neurogenetic Institute, Keck School of Medicine, University of Southern California, Los Angeles, California, United States of America; 3 Department of Psychiatry, Keck School of Medicine, University of Southern California, Los Angeles, California, United States of America; 4 Division of Bioinformatics, Department of Preventive Medicine, Keck School of Medicine, University of Southern California, Los Angeles, California, United States of America; Peking University Health Science Center, China

## Abstract

**Background:**

Pituitary adenomas (PAs) are commonly occurring neoplasms with diverse endocrine and neurological effects. Although somatic gene mutations are uncommon in sporadic PAs, recent studies lend support to epigenetic modification as a potential cause of tumorigenesis and tumor progression.

**Methods:**

A systematic literature review of the PubMed and Google Scholar databases was conducted to identify abstracts (n=1,082) pertaining to key targets and mechanisms implicated in epigenetic dysregulation of PAs published between 1993-2013. Data regarding histopathological subtype, target genes, mode of epigenetic modification, and clinical correlation were recorded and analyzed.

**Results:**

Of the 47 that studies met inclusion criteria and focused on epigenomic assessment of PAs, only 2 were genome-scale analyses. Current evidence supports epigenetic alteration in at least 24 PA genes, which were categorized into four groups based on function and epigenetic alteration: 1) Sixteen tumor suppressor genes silenced via DNA methylation; 2) Two oncogenes overexpressed via histone acetylation and hypomethylation; 3) Three imprinted genes with selective allelic silencing; and 4) One epigenome writer inducing abnormal genome-scale activity and 5) Two transcription regulators indirectly modifying the genome. Of these, 5 genes (*CDKN2A, GADD45y, FGFR2*, caspase-8, and *PTAG*) showed particular susceptibility to epigenetic modification, with abnormal DNA methylation in >50% of PA samples. Several genes displayed correlations between epigenetic modification and clinically relevant parameters, including invasiveness (*CDKN2A; DAPK; Rb1*), sex (*MAGE-A3*), tumor size (*GNAS1*), and histopathological subtype (*CDKN2A; MEG3; p27; RASSF1A; Rb1*).

**Conclusions:**

Epigenetic modification of selected PA genes may play a key role in tumorigenesis and progression, which may translate into important diagnostic and therapeutic applications.

## Introduction

Pituitary adenomas (PAs) are among the most commonly occurring intracranial neoplasms, representing 10-15% of newly diagnosed intracranial tumors. Although they are predominately benign tumors, PAs may result in malignant endocrinopathies caused by hormonal hypersecretion and/or or tumor mass effect resulting in hypopituitarism and visual dysfunction. In comparison to other solid tumors, PAs rarely become malignant, with pituitary carcinoma comprising 0.1% of all pituitary tumors[1,2]. 

Similarly, PAs display a comparative paucity of somatic gene mutations in comparison to other neoplasms. Instead, PAs demonstrate a propensity for altered genetic function via epigenetic modification and variable gene expression. Epigenetic modification is a global term describing a variety of molecular processes that may affect gene expression without altering the underlying DNA base sequence. At least five major epigenetic mechanisms exist that may result in modified gene expression: 1) DNA methylation, 2) Histone modification, 3) Gene imprinting, 4) Epigenome writers, and 5) Transcription regulators. Although these five mechanisms comprise a critical part of normal cellular function, they have only recently been implicated in driving tumorigenesis and progression of various neoplastic conditions. A careful balance between these epigenetic mechanisms is required to maintain normal cellular function and inhibit uncontrolled cell growth associated with neoplasia.

Elucidation of the mechanisms underlying tumorigenesis and transformation from benign to invasive/atypical PA represents a major challenge to the current understanding of these tumors, and may have important implications for diagnostic molecular classification, prognosis regarding tumor progression and recurrence, guiding adjuvant treatments such as radiation therapy, and identifying potential targets for future therapies. In an effort to consolidate prior research regarding this subject, we conducted a systematic literature review to identify studies pertaining to target genes and mechanisms frequently involved in epigenetic modification of PAs. In the current review, we summarize and discuss the role of epigenetic modification in the tumorigenesis, functional classification, invasion, and progression of PAs. 

## Materials and Methods

### Ethics Statement

This study was conducted according to the Helsinki human subject doctrine and approved by the USC Ethics Committee/Institutional Review Board.

A PubMed and Google Scholar database search was conducted to identify all studies pertaining to epigenomic analysis of PAs published in the English language between 1993-2012. The 2009 PRISMA statement was used as a guideline for performing the review [3]. The following search terms were utilized: pituitary adenoma, epigenetic, methylation, CPG island, histone, acetylation, chromatin remodeling, gene silencing, and imprinting. Studies focusing on epigenetic modification of PAs in humans or animals were included. Studies focusing strictly on somatic gene mutations or gene expression without epigenetic analysis were excluded. In addition, references of all identified articles were searched for additional studies meeting inclusion criteria. Additional protocols for systematic analyses of PA epigenetic mechanisms were also reviewed and modified to meet the objectives of the current review[4,5]. Articles involving epigenetic analysis of pituitary adenoma tissue performed as candidate gene studies, genome-scale studies, and review articles were included and designated as such. 

**Figure 1:**
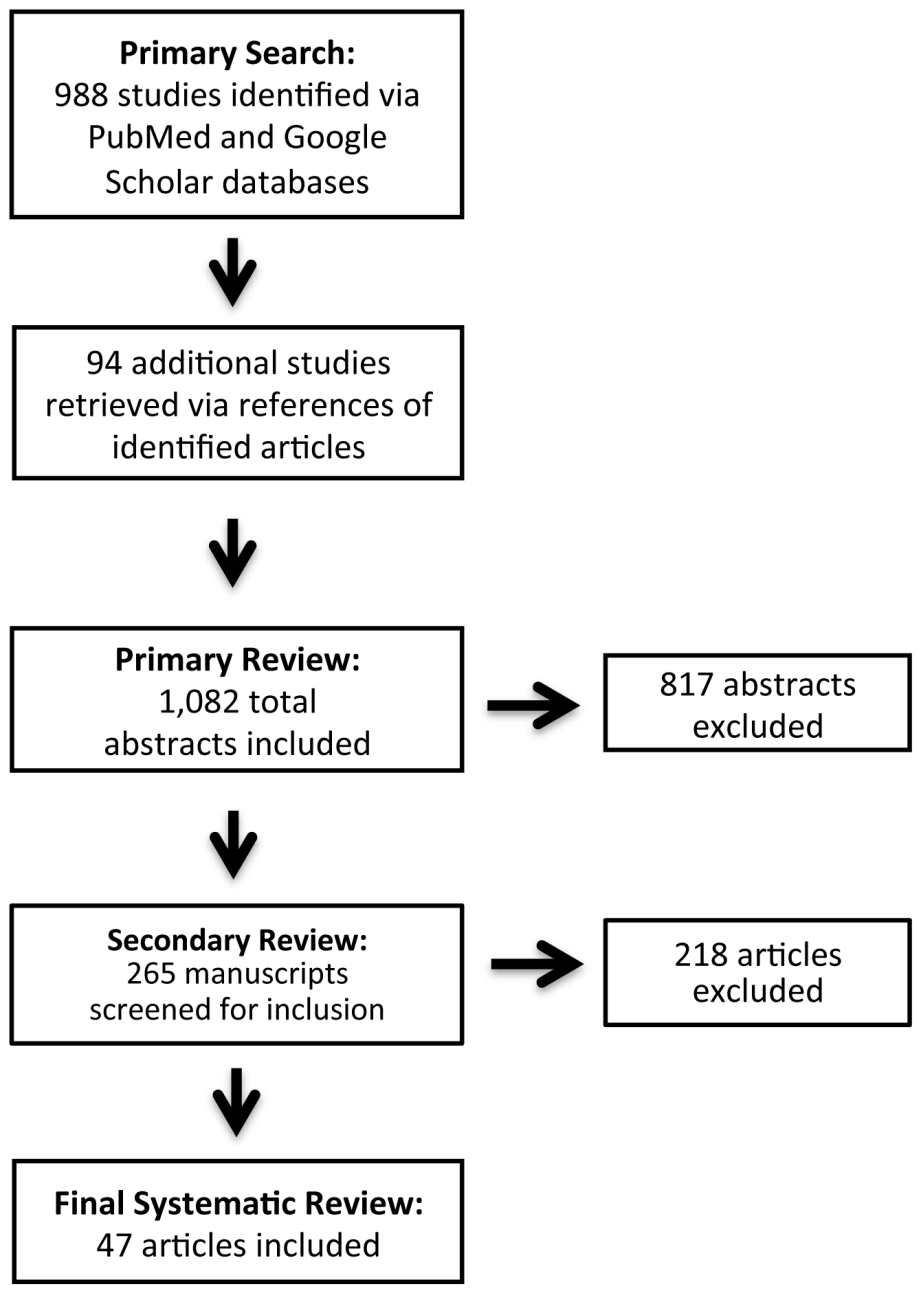
Flow diagram depicting systematic review search results according to phases.

A primary search yielded 1,082 studies that underwent initial abstract review (Figure *1*), of which 817 studies were excluded. Of the 265 remaining studies that underwent in-depth secondary review, 47 met final inclusion/exclusion criteria and were included in the analysis. Data including type of analysis, key genes and mechanisms, sample size, and clinical correlations were extracted, entered into an electronic database, and reviewed. 

Where applicable, pooled data regarding clinical correlations and PA tumor subtypes from the 47 included studies were compiled. Due to differences in the technological platforms used to detect methylation and differences in data reporting, not all studies were directly comparable. Assessment of potential for bias in individual studies was not possible and therefore was not performed. A majority of studies reported a percentage of methylated tumors and were thus easily comparable. Studies assessing other genes, especially imprinted PA genes, relied on more complex methylation mapping requiring a comparison of methylation percentages of several different CpG islands within a promoter region. As a result, the various studies on imprinted genes were not completely analogous and are reported separately. 

## Results

Forty-seven studies were identified that focused on epigenomic assessment of PAs. Of these, only 2 studies were genome-scale analyses, 27 were candidate gene studies, and 18 were review articles. Epigenetic regulation of PAs was classified according to two major factors, the mechanism of epigenetic regulation (5 basic mechanisms) and the type of gene regulated. Although a detailed description of each gene/mechanism is beyond the scope of this review, representative mechanisms of the five major modalities of epigenetic regulation or dysregulation are described below. Furthermore, Table *1* outlines the 24 known epigenetically-modified genes implicated in PAs to date, categorized according to the gene’s basic role in tumorigenesis: (1) Tumor Suppressor Genes (TSGs); (2) Oncogenes; (3) Imprinted Genes; (4) Epigenome Writers; and (5) Transcription Regulators. 

**Table 1:** Classification of 24 genes found to be epigenetically-modified in pituitary adenomas, according to gene type.

**Gene Type**	**Gene Name**	**Gene Symbol/Product**	**References**
**Tumor Suppressor Genes (n=16)**	Cyclin-dependent Kinase Inhibitor 2A	*CDKN2A*/p16^INK4a^	[16,18,19,21–27,29,36,37,64]
	Retinoblastoma	*RB1*/pRb	[18,24,31–36,40,41,60,65]
	Cyclin-dependent Kinase Inhibitory 1A and B	*CDKN1A and B*/p21 and p27	[37,39,61]
	Death Associated Protein kinase	DAP kinase	[15,18,60]
	p73 Gene	p73	[39,60]
	Cyclin-dependent Kinase Inhibitor 2A Alternate Reading Frame	*CDKN2A*/p14^ARF^	[39,60]
	Growth Arrest and DNA damage-inducible protein	*GADD45γ*	[59,66,67]
	Fibroblast growth factor receptor 2	*FGFR2*	[6,43,61]
	E-cadherins	*E-cadherins*	[65,66]
	Capase-8	*Capase-8*	60
	Ras Association Domain Family 1A	*RASSF1A*	68
	Rhomboid domain-containing protein 3 (RHBDD3)/Pituitary Tumor Apoptosis Gene (PTAG)	*RHBDD3* and *PTAG*	59
	Tissue Inhibitor of Metalloproteinase 3	*TIMP-3*	60
	O(6)-Methylguanin-DNA methyltransferase	*MGMT*	60
	Thrombospondin-1	*TSP-1*	60
	S100A10	*S100A10/*p11	[5,7]
**Oncogenes (n=2)**	Melanoma-associated Antigen 3	*MAGEA3*	[6,43,61]
	Pituitary Tumor Transforming Gene	*PTTG*	[42,45–47]
**Imprinted Genes (n=3)**	Guanin nucleotide-binding protein G_s_α subunit	*GNAS1*	[11,47,51]
	Neuronatin	*NNAT*	[7,71]
	Maternal Imprinted Gene 3	*MEG3*	[50,51]
**Epigenome Modifiers (n=3)**	DNA Methyltransferase 3b	*DNMT3b*	[7,61]
**Transcription Regulators** (**n=2**)	Ikaros	*Ik*	[10,44,54,66,70]
	High Mobility Group A2*	HMGA2*	53

### Basic Mechanisms of Epigenetic Modification

#### (1) DNA methylation

DNA methylation is the most frequently studied epigenetic phenomenon, and is a necessary physiological mechanism utilized to functionally ‘silence’ genes by hindering transcriptional machinery’s access to DNA via alteration of CpG dinucleotides. Roughly 80% of CpG dinucleotides in the human genome are subject to methylation changes throughout life, and nearly 70% of CpG islands are methylated at any given time, indicating the widespread regulatory scope of DNA methylation [6]. Although most unmethylated CpG dinucleotides are located in CpG-dense promoter regions called CpG islands [7], and methylation of these regions functionally silences the promoter and corresponding gene, in more recent years it has come to be known that global methylation of non-CPG island dinucleotide regions may play as critical a role in epigenetic modulation[8,9]. 

#### (2) Histone modification

Chromatin is a dense assembly of DNA essential for packing the genome within the confined space of the nucleus. To accomplish this, DNA is coiled twice around octomeric proteins called histones (H1-4), which facilitate a high degree of DNA organization and control accessibility to the genome [6]. Two main processes, histone acetylation and methylation, alter the accessibility of DNA to either facilitate or diminish translation. Acetylation of lysine (k) residues on histone H4 and methylation of lysine 9 (K9) on H3 restrict access to promoter regions, whereas acetylation on K9 and K14 of H3 and methylation on K4 of H3 activate gene promoter regions. Although direct gene methylation is the most thoroughly investigated epigenetic process, histone alteration likely plays an equally important role regulating the epigenome. Indeed, direct functional links between DNA and histone methylation indicate that the two are functionally dependent, with histone methylation being a prerequisite for DNA methylation [6,10]. Together, DNA and histone methylation are necessary processes contributing to a stable genome.

#### (3) Gene imprinting

Imprinting is a common epigenetic mechanism whereby an allele of a gene is inactivated through complete CpG island methylation. This process is also a normal developmental phenomenon, and allows one parental gene to fully dictate the phenotype of an offspring [11]. Unlike normal genes, an imprinted gene will show methylation rates in excess of 50%, since one of the two alleles is completely methylated. This may render the phenotypic expression of the gene susceptible to alteration because any modification to the functional allele, such as methylation-induced silencing or somatic mutation, will alter gene expression.

#### (4) Epigenome Modifiers

Certain proteins, such as DNA methyltransferases (DNMTs), can directly alter the entire epigenome and DNA accessibility through the aforementioned mechanisms. These proteins are normally responsible for establishing and maintaining the epigenome through performing DNA and histone modifications. Generally, epigenome modifiers perform three broad functions, including writing the epigenome, reading it, and editing its content [7]. Unlike the other mechanisms, epigenome controllers can cause widespread epigenetic changes that increase or decrease the overall expression of myriad genes, rather than one individual gene.

#### (5) Transcription Regulators

Transcription regulators are an essential part of genetic regulation that control gene expression levels. Similar to epigenome modifiers, transcription regulators can become epigenetically modified and alter the expression of genes other than themselves or downstream modifiers.

### Epigenetic Modification by Gene Subtype

#### 1) Tumor Suppressor Gene Inactivation: The Example of CDKN2A And Rb1

A tumor suppressor gene (TSG) inhibits a cell line from progression towards neoplasia, often by encoding a group of regulatory proteins that inhibit cell cycle progression and/or promote apoptosis to achieve cell quiescence. TSGs may lead to tumorigenesis from loss of function event(s) that require both copies of the gene to become inactivated, or acquire “two hits” [12]. Three principal mechanisms exist for the loss of function of a TSG: (i) Loss of one allele and an inactivating mutation in the other allele resulting in loss of heterozygosity (LOH); (ii) Homozygous deletion of both alleles leading to loss of function; and (iii) Epigenetic changes resulting in gene silencing, such as the methylation of CpG islands [13,14]. Depending on a particular tumor subtype, each mechanism may play a more or less critical role in tumorigenesis. Because TSGs are normally unmethylated at CpG island sites, however, they provide ripe targets for potential aberrant methylation resulting in gene silencing [15].

In our review, 16 TSGs in PAs were reported to undergo frequent silencing via epigenetic modification (Table *2*). All 16 genes had reduced expression due to an inactivating event, and subsequent inhibition of the gene’s tumor suppressing function. A majority of these TSGs (11/16) act via modulation of apoptosis and cell cycle progression. Two major human TSG pathways, the *CDKN2A*(p16)/*Rb1*(pRb) and *p53* pathways, act through these very mechanisms to inhibit cell proliferation and neoplastic growth [16,65]. The *CDKN2A*(p16)/*Rb1*(pRb) pathway normally inhibits the G_1_ to S cell cycle phase transition, and the p53 pathway both regulates cell cycle progression and promotes apoptosis. Both pathways limit growth potential and tumorigenesis by decreasing cell line viability and uncontrolled growth. These pathways are commonly affected in many cancer types, and were implicated in 8 of 16 TSGs in the current study, either directly or through downstream modifiers [17]. 

**Table 2:** Sixteen tumor suppressor genes (TSGs) affected via DNA methylation (all) and histone acetylation (*).

**Gene**	**Function**	**Pathway Altered**
*CDKN2A/p16^INK4a^*	Cell cycle regulation (G1 to S phase transition)	CDKN2A/Rb1
*Rb1/pRb*	Cell cycle regulation (G1 to S phase transition)	CDKN2A/Rb1
*p21 and p27*	Cell cycle regulation (G1 to S phase transition)	p53
*DAP kinase*	Apoptosis (programmed cell death mediated by p19ARF)	p53
*p73*	Apoptosis (functionally homologous to p53)	p53
*p14^ARF^*	(1) Apoptosis; (2) Cell cycle regulation	p53
*GADD45γ*	(1) DNA repair; (2) Cell cycle regulation; (3) Apoptosis; (4) p53 stability; (5) Global DNA hypermethylation	(1) p53; (2) AID/Apobec-1; (3) JNK pathway
FGFR2*	Apoptosis and cell cycle regulation (through p53)	p53
*E-cadherins*	Fibrous bodies	Unknown
*Capase-8*	Apoptosis; ECM degradation	Death receptors 4 and 5
*RASSF1A*	Cell cycle regulation and apoptosis	(1) Microtubules; (2) Cyclin D1; (3) Extrinsic apoptosis pathway
*RHBDD)/PTAG*	Apoptosis	Mitochondrial membrane function
*TIMP-3*	Apoptosis	Death receptor apoptosis
*MGMT*	DNA repair	DNA repair
*TSP-1*	Angiogenesis	CD36
*S100A10*	ECM degradation	Inflammation

The *CDKN2A*(p16)/*Rb1*(pRb) pathway, altered in up to 90% of PAs, provides a classic example of an epigenetically-controlled TSG [16]. This pathway promotes cell quiescence by regulating phosphorylation of the downstream effector pRb [18]. Initially, CDK4/6 and cyclin D1 (cycD1) form a productive interaction that leads to CDK4/6 phosphorylating pRb [19]. This interaction is regulated by p16, which binds to CDK4/6 to inhibit the CDK4/6-cycD1 interaction and, in turn, the phosphorylation of pRb. In the hyperphosphorylated conformation, pRb releases transcription factors (TFs) such as E2F that facilitate cell cycle progression. The hypophosphorylated pRb conformation, in contrast, binds to the TF to promote cell quiescence. Based on their influence on TFs promoting cell cycle progression, both p16 and pRb may aptly be viewed as “cell cycle brakes” that carefully regulate cell proliferation, as demonstrated by the fact that 100% of *Rb1* transgenic knockout mice develop pituitary tumors [20]. The inactivating events in these 16 epigenetically altered TSGs result in diminished gene expression and protein production. For example, p16 expression is strongly diminished (0-17%) in the majority of studies reviewed [16,21–24]. 

Epigenetic modification is likely to be the predominant mechanism involved in the development of PAs, and provides a compelling mechanism for inactivation of the 16 TSGs reviewed in this study. In PAs, the *CDKN2A* gene is almost exclusively inactivated via methylation, with inactivating mutations (LOH) or homozygous deletion accounting for only 0-15% of *CDKN2A* dysfunction [16,19,21,23,25,26]. Methylation of the *CDKN2A* gene’s CpG islands in sporadic PAs was detectable in 34–90% of tumors, and notably absent in normal pituitary gland samples [16,21,23,27–29]. Furthermore, demethylating agents such as 5 aza-2-deoxycytidine induced re-expression of p16 in cell lines with widespread methylation, supporting a causal link between methylation and inactivation in PAs [30].

Although a review of mechanisms involved in silencing of the *Rb1* gene shows a more balanced landscape of inactivation, epigenetic mechanisms nevertheless predominate [31–33]. Seeman et al. found that 60% of PAs that failed to express pRb were methylated [16]. In this study, however, primary mutations still played a role in PA development, with 30% of pRb-silent PAs displaying inactivating mutations also correlating with tumor invasiveness. Although this correlation could be explained by general genetic instability in more aggressive tumors, both LOH and epigenetic modifications are likely to play a prominent role inactivating *Rb1* [34–38].

The inactivation of the *CDKN2A* and *Rb1* pathways showed a high degree of specificity and efficiency. Methylation of the *CDKN2A* gene is a specific phenomenon and not indicative of widespread epigenetic changes, based on high rates of methylation of *p15* (another TSG), but not the neighboring *p14* gene [39]. Furthermore, duplicate inactivations (i.e. concurrent LOH and methylation of the same *Rb1* gene) are rare and mutually exclusive events in PAs, and appear to provide no additional growth benefit to neoplastic cells [16]. Similarly, parallel inactivation of multiple pathways affecting the same downstream modifier also occurs infrequently. *CDKN2A* and *Rb1* inactivation are often mutually exclusive events and occur concomitantly in less than 12% of cases (5/45) [24,28]. This discriminative relationship, coupled with the gene specific inactivation of *CDKN2A*, may lead to differences in the clinical profile of specific tumors. For example, loss of pRb expression is tumor subtype-specific and inversely related to p16 expression: 31% of somatotropinomas and 17% of nonfunctional PAs failed to express pRb, whereas 25% of somatotropinomas and 66% of nonfunctional PAs showed altered p16 expression [28,36] On the other hand, this discrimination between inactivating various pathways applies only to those with similar downstream effectors. For example, the *Rb1* and *p53* pathways promote cell survival through different mechanisms and frequently show strong coincidence. The *p53* regulators p14^ARF^, p21^Wafl/Cip1^, and p73 showed complete overlap in all but one PA with the more common *Rb1* pathway aberrations [39]. This overlap suggests that PAs are likely to acquire survival-enhancing genetic alterations of varying downstream pathways.

In summary, TSGs promote cell line neoplasia through a complex interaction of pathways, often involving gene silencing via DNA methylation. Although LOH, deletion, and methylation all may inactivate TSGs to varying degrees, these mechanisms rarely occur concordantly in the same TSG. Pathways with identical downstream effectors (i.e. *CDKN2A* and *Rb1*) are usually individually inactivated in individual PAs, whereas pathways with varying effects (i.e. *Rb1* and *p53*) are frequently inactivated concordantly. Taken together, TSG inactivation in PAs comprises a group of efficient mechanisms that accumulate growth-enhancing effects without amassing excess or unproductive genetic modifications.

#### 2) Pro-Oncogenes and Histone Acetylation

In general, pro-oncogenes produce the opposite effects of TSGs by facilitating cell-cycle progression, maintaining chromosomal stability, and inducing aneuploidy [40]. To date, only 2 modified pro-oncogenes have been reported in PAs (Table *3*). Unlike the methylation-induced silencing generally observed in TSGs, these genes show increased expression levels. For example, *MAGEA3* increases gene expression through interactions with *FGFR2* to produce gene promoter hypomethylation, which decreases inhibition of transcription and increases gene expression [41,42]. Pituitary Tumor-Transforming Gene (PTTG) is also overexpressed in PAs as compared to normal pituitary gland as a result of histone acetylation [43,44]. The histone acetyltransferase (HAT) p300 upregulates histone H3 acetylation at the *PTTG* promoter [45], and directly promotes increased mRNA expression and protein levels, thereby allowing PTTG to exhibit its deleterious effects through c-Myc and FGF2 function. Not unexpectedly, the PA epigenome displays a global increase in histone acetylation as compared to normal pituitary cells [46]. Although histone modification is less well understood than DNA methylation, it may play an equally important and dynamic role in epigenetic regulation of PAs.

**Table 3:** Alternative, non-methylation based methods of epigenetic regulation (including histone acetylation, gene imprinting, epigenetic writers, and transcription regulators) identified in 8 additional PA genes.

**Gene**	**Type**	**Function**	**Pathway Altered**	**Effect on Gene Expression**	**Mode of Activation or Inactivation**
*MAGEA3*	Oncogene	(1) Apoptosis; (2) Cell cycle regulation	p53	Increase	Hypomethylation
*PTTG*	Oncogene	Cell cycle progression and chromosome stability	(1) c-Myc; (2) FGF2	Increase	Histone acetylation
*GNAS1*	Imprinted Gene	G-protein function; oncogene	G-protein function	Expression of mutated gene	Imprinting relaxation
*Neuronatin (NNAT)*	Imprinted Gene	Cell cycle regulation and apoptosis	Uncertain	Decrease	Methylation of non-imprinted gene
*MEG3*	Imprinted Gene	(1) Apoptosis; (2) Cell cycle regulation	p53	Decrease	Methylation of non-imprinted gene
*DNMT3b*	Epigenetic Writer	Increased methylation	De novo methylase	Increase	Hypomethylation and histone modification
*Ikaros*	Transcription Regulator	(1) Apoptosis; (2) Hormone gene expression	(1) Bcl-XL apoptotic pathway; (2) GH and PRL gene expression	Alternate spliced isoforms	Increased methylation and alternately spliced isoforms Ik1, Ik2/3, and Ik6
HMGA2*	Transcription Regulator	Cell cycle regulation	E2F-pRb complex	Increase	None discovered

#### 3) Imprinted Genes: The case of MEG3

Gene imprinting is a specific subtype of methylation whereby one parental allele of a gene is inactivated through high levels of methylation. This can cause tumorigenesis through two major mechanisms. First, some PA genes (i.e. *GNAS1*) demonstrate relaxed imprinting, which results in induced overexpression of the gene product or allows the expression of a previously silenced gene mutation (Table *3*) [11,47]. For other imprinted genes, having only one functional allele renders the gene product susceptible to alterations such as mutation or increased methylation. 

Maternal Imprinted Gene 3 (*MEG3*) is an imprinted gene that is modified in PAs via DNA methylation. In normal pituitary cells, *MEG3* retains strong expression, functions as a powerful growth suppressor [48,49], and primarily acts by increasing *p53* expression and modifying its transcriptional activation [50]. Loss of *MEG3* leads to decreased expression of *p53*, increased cell survival through activated cell cycle progression, and decreased apoptosis. From a structural standpoint, *MEG3* contains two CpG-rich 5’-flanking regions, making it an ideal candidate for methylation, as would be expected for an imprinted gene. In normal pituitary gland, *MEG3* displays approximately 50% methylation rates (consistent with an imprinted gene). The *MEG3* promoter regions in PAs with MEG3 deficiency showed significantly increased (>50%) methylation of CpG islands. The increased accessibility of the CpG islands that are generally susceptible to imprinting may also facilitate methylation-induced inactivation of the functioning *MEG3* allele. In a sample of 13 nonfunctional PAs, *MEG3*-deficient PAs displayed no evidence of LOH or other genetic mutations [49]. 

#### 4) Epigenetic Modifiers: DNMT3b Rewriting the Epigenetic Code

The regulatory machinery that controls epigenetic modification can itself become altered in PAs. The epigenome writer DNMT3b, a protein that generates 5-methylcytosine by adding a methyl group directly to a cytosine base, is upregulated in PAs via histone modification. This upregulation may lead to a methylation-induced silencing of other genes predominately featured in PAs, such as *Rb*, *p21*, and *p27* [7], which will subsequently lead to increased cell proliferation. DNMT3b functions as a putative mediator of the epigenome that affects numerous genes.

#### 5) Transcription Regulators: Ikaros Passes on Epigenetic Modifications

Transcription regulators alter gene expression in PAs by changing gene transcription rates. Ikaros is a family of zinc-finger DNA-binding proteins that is part of a complex chromatin-remodeling network [54]. As a family, wild-type Ikaros inhibits GH mRNA and stimulates PRL mRNA through histone deacetylation and acetylation, respectively. As a family, Ikaros is susceptible to methylation of CpG islands in one of its exons. Through an unknown mechanism, PAs specifically overexpress a dominant negative alternatively spliced form of Ikaros [52,66,69]. This spliced form subsequently upregulates an anti-apoptotic signal through altering gene accessibility. Due to the Ikaros family’s susceptibility to epigenetic modification, demethylating agents may potentially play a role in treating PAs with altered Ikaros function.

## Correlation to Clinical Characteristics

Combined with knowledge of the mechanisms underlying epigenetic aberration, understanding the clinical implications and downstream effects of these modifications may facilitate more targeted treatment of PAs. Pooled data from the 47 reviewed studies regarding associations with functional PA tumor subtypes and clinical characteristics are highlighted in Tables *S1* and *S2*, respectively. Although sample sizes in most PA epigenetic studies are small, and the frequency estimates lack a high level of accuracy, they nevertheless provide useful ranges for epigenetic effects associated with PA tumorigenesis. Some of the most important prognostic characteristics for determining effective PA treatment include histopathological subtype, degree of tumor invasion (i.e. Knosp grade), World Health Organization (WHO) grade, and tumor size, all of which are also associated with extent of surgical resection and likelihood of subsequent tumor recurrence/progression [1,53–56]. Furthermore, although the roles of some epigenetically modified genes (including *HMGA2* and *PTTG*) have yet to be validated in human PA samples, they are described in mouse models of human pituitary cell lines [43,51,57]. Despite these limitations, epigenetic regulation of particular genes has been correlated with specific clinical characteristics, potentially rendering selected PAs more susceptible to epigenetic therapies.

Overall, methylation rates of several PA genes varied across the tumor samples studied. The TSG genes reviewed can be classified according to the degree of methylation: Frequently methylated genes (i.e. *CDKN2A, GADD45y, FGFR2, caspase-8, and PTAG*) demonstrate methylation in over 50% of PAs; moderately methylated genes (i.e. *TSP-1, RASSF1A, Rb1, p73, MGMT, and E-cadherin*) demonstrate methylation in 20-50% of PAs; and infrequently methylated genes (i.e. *p14, DAP kinase, TIMP3, p21, and p27*) are methylated in <20% of PAs [39,40,58–60,67,68,70]. These variable rates of methylation may reflect a variety of factors. For some genes with large sample sizes available (i.e. *CDKN2A*, n =393), the percentage of PAs with gene methylation is relatively well established. On the other hand, genes such as *TIMP3* had much smaller reported sample sizes (n=35) that, combined with the infrequent methylation rates observed, may reflect random variation rather than the true tumor methylation percentage. Furthermore, as discussed previously, methylation is only one avenue involved in gene silencing. 

Seven genes were reported to demonstrate an association between degree of methylation and PA invasiveness. When comparing the percentage of methylated tumors among invasive versus non-invasive PAs, *Rb1* and *DAPkinase* showed significant differences, whereas no differences in *E-cadherins, GADD45y, RASSF1A*, and *PTAG* were detected. The relationship between epigenetic *CDKN2A* silencing and selected clinical features of PAs is not as clearly understood. Although earlier reports did not identify a correlation between *CDKN2A* methylation and tumor volume, invasiveness, recurrence, or malignancy, more recent studies have associated *CDKN2A* methylation with tumor volume, grade, and patient age [16,25,27,29,36]. The correlation between tumor volume and *CDKN2A* silencing makes intuitive sense, as the loss of the p16 G_1_/S checkpoint could result in a higher tumor proliferation rate. Indeed, much higher rates of *CDKN2A* methylation have been reported in pituitary macroadenomas (77/118, 65.3%) than in pituitary microadenomas (4/30, 13.3%, p<0.001). Although the exact timing of *CDKN2A* methylation in PA development remains uncertain, this modification appears to be somehow implicated in PA tumorigenesis and progression. In the case of *Rb1* and *DAP kinase*, however, the correlation with invasion suggests that methylation is likely to be a later event in tumorigenesis. 

Other clinical characteristics, such as patient age and sex, appear to be correlated less with methylation status. A handful of studies reported a significant relationship between patient age and methylation status, although this could also reflect the general trend that global methylation is correlated with age [61]. Two genes, *CDKN2A* and *MAGE-A3*, showed varying methylation rates in PAs between the two sexes [16,21,23,25,27,29,36,41]. 

Although specific histopathological PA subtypes also show differential methylation patterns, for most genes the relationship between DNA methylation and histopathological subtype has not been elucidated [18]. *Rb1* and *RASSF1A*, for example, are preferentially expressed in functional PAs, *MEG3* is most frequently methylated in gonadotropinomas, and *p27* is predominantly methylated in NFAs. Finally, several other genes show decreased methylation in specific histopathological PA subtypes, including *CDKN2A* in GH and ACTH adenomas, *RASSF1A* in FSH/LH adenomas, and *p27* in ACTH adenomas. CDKN2A is rarely methylated in GH and ACTH adenomas, and instead shows higher levels of mutations and LOH at the CDKN2A locus in prolactinoma and nonfunctional subtypes (p<0.002) [16,25,27,29,62]. *E-cadherin* provides a unique example of preferential epigenetic regulation in a particular histopathological PA subtype, and according to one study was methylated in 23% of GH-adenomas (6/26), but failed to demonstrate methylation in any other PA subtype [63,64]. 

## Discussion

Perhaps the most important clinical aspect of this research lies in its potential to develop drugs that can modify epimutations. Although the majority of symptomatic non-prolactinoma PAs are currently treated via surgical resection, important advances will soon likely facilitate improved medical treatment for PAs outside of the well-established dopamine analogs for prolactinomas. Unlike gene mutations, epigenetic modifications are typically reversible, and epigenetically altered PAs could therefore be potentially cured with drug therapy. Two major strategies exist for targeting epigenetic dysregulation of PAs: (1) Alteration of genome-scale epigenetic processes; and (2) Alteration of methylation or acetylation of specific genes [4]. Several drugs (i.e. DNMT inhibitors) are already known to accomplish the former goal, and are currently used to treat several disorders including myelodysplastic syndrome. Likewise, HDAC inhibitors affect deacetylation to provide the opposite effect of DNMT inhibitors. No agents, however, are yet known to target epigenetic processes at specific gene loci. Furthermore, both drug classes affect the entire genome, and therefore hold the potential for causing significant adverse systemic effects. Narrowing therapeutic specificity is a major challenge that lies ahead and will be required for successfully targeting epigenetic regulation of PAs via pharmacological means. 

One focused goal of future research may involve identifying genes uniquely modified in PAs, or genes essential to the tumorigenesis of PAs. For example, in gliomas, *MGMT* methylation patterns are predictive and may help guide treatment options [71]. Many of the genes mentioned in this review, including *p16, pRb*, and *DAPK* are epigenetically altered in a number of cancers. Several of the other aforementioned genes, however, have oncogenic effects that are distinct in PAs. For example, Ikaros has a noteworthy role regulating neuro-hormone expression in the pituitary and shows altered expression in PAs. Likewise, *PTAG* is a proapoptotic gene that shows decreased expression in PAs. Initially discovered in PAs, this gene has since been implicated in other tumor types [59]. One major goal is to find candidate genes that can become targets for epigenetic treatment or act as prognostic markers (e.g. *ZAP70* in chronic lymphocytic leukemia), to be able to accurately predict treatment responses [72].

Although the current study highlights many of the epigenetic processes implicated in PA tumorigenesis, there are many limitations of this review methodology. The potential exists for selection bias from each of the contributing studies, as well as publication bias from unpublished and potentially negative studies. Furthermore, the heterogeneity in genomics techniques and reporting styles utilized over the duration of included studies (targeted versus genome-scale, various sequencing platforms, sample size variations, etc.) also limit the overall interpretation of these results. Nevertheless, the genes and mechanisms identified and underscored in this review may serve as a basic platform summarizing the wealth of information related to epigenetic regulation of PAs that has been gleaned over the past several decades.

## Conclusions

Recent advances in our understanding of epigenomic regulation have elucidated some of the mechanisms underlying PA tumorigenesis and progression. PA genes have a propensity for inactivation via epigenetic modification, as compared to primary mutagenic events in somatic genes. Methylation-induced gene silencing is a well-established epigenetic phenomenon, whereas the role of histone acetylation in PAs is still being investigated. Improved understanding of the epigenetic mechanisms regulating gene expression of PAs may facilitate targeted development of therapeutic agents affecting the phenotypical behavior of these tumors.

## Supporting Information

Table S1 **Epigenetic gene regulation (DNA methylation) according to PA histopathological subtype.**
(DOCX)

Table S2 **Epigenetic gene regulation (DNA methylation) associated with clinical and tumor characteristics (age, sex, invasiveness).**
(DOCX)

Checklist S1 **PRISMA Checklist for systematic reviews.**
(DOC)
