# Supplementary material for: The Role of Epigenetic Modification in Tumorigenesis and Progression of Pituitary Adenomas: A Systematic Review of the Literature
Source: PLoS One. 2013 Dec 18;8(12):e82619. doi: 10.1371/journal.pone.0082619 (PMC3867353; doi:10.1371/journal.pone.0082619)
Supplement: Table S1 — Epigenetic gene regulation (DNA methylation) according to PA histopathological subtype. (DOCX) [file pone.0082619.s001.docx]

| **Gene** | **References** | **Nonfunctional**  **PAs** | | **All Functional PAs** | **Prolactinomas** | | | **GH-adenomas** | | **ACTH-**  **adenomas** | | | **FSH/LH adenomas** | | | |  |  |  |
| --- | --- | --- | --- | --- | --- | --- | --- | --- | --- | --- | --- | --- | --- | --- | --- | --- | --- | --- | --- |
| *Ikaros** | 8, 52 | 4/5 (80.0%) | | 6/10 (60.0%) | 4/6 (66.6%) | | | 2/4 (50.0%) | | - | | | 4/5 (80.0%) | | | |  |  |  |
| *GADD45y* | 57 | 8/17 (47.1%) | | 10/16 (62.5%) | 1/1 (100.0%) | | | 9/15 (60.0%) | | - | | | - | | | |  |  |  |
| *CDKN2A* | 14, 19, 21, 23, 25, 26, 27, 37, 65 | 112/160 (70.0%) | | 70/137 (51.1%) | 22/30 (73.3%) | | | 13/57 (22.8%) | | 23/50 (46.0%) | | | 6/11 (54.5%) | | | |  |  |  |
| *FGFR2* | 41 | 3/5 (60.0%) | | 6/13 (46.2%) | 4/6 (66.6%) | | | 2/4 (50.0%) | | 0/2 (0.0%) | | | 3/5 (60.0%) | | | |  |  |  |
| *Caspase 8* | 58 | 13/23 (56.5%) | | - | 4/7 (57.1%) | | | 2/4 (50.0%) | | 0/1 (0.0%) | | | - | | | |  |  |  |
| *PTAG* | 57 | 9/17 (52.9%) | | 9/18 (50.0%) | - | | | 9/18 (50.0%) | | - | | | - | | | |  |  |  |
| *TSP-1* | 58 | 9/23 (39.1%) | | 6/12 (50.0%) | 3/7 (42.9%) | | | 2/4 (50.0%) | | 1/1 (100.0%) | | | - | | | |  |  |  |
| *RASSF1A* | 58 | 6/21 (28.6%) | | 14/31 (45.2%) | 5/11 (45.5%) | | | 7/13 (53.8%) | | 2/4 (50.0%) | | | - | | | |  |  |  |
| *Rb1* | 23, 37, 58, 65 | 23/78 (29.5%) | | 27/63 (42.9%) | 7/25 (28.0%) | | | 10/34 (29.4%) | | 0/1 (0.0%) | | | - | | | |  |  |  |
| *p73* | 58 | 6/23 (26.1%) | | 4/12 (33.3%) | 3/7 (42.9%) | | | 1/4 (25.0%) | | 0/1 (0.0%) | | | - | | | |  |  |  |
| *MGMT* | 58 | 5/23 (21.7%) | | 3/12 (25.0%) | 1/7 (14.3%) | | | 2/4 (50.0%) | | 0/1 (0.0%) | | | - | | | |  |  |  |
| *p14* | 58 | 3/23 (13.0%) | | 3/12 (25.0%) | 2/7 (28.6%) | | | 1/4 (25.0%) | | 0/1 (0.0%) | | | - | | | |  |  |  |
| *DAP Kinase* | 13, 16, 58 | 3/31 (9.7%) | | 6/23 (26.1%) | 0/7 (0.0%) | | | 1/3 (33.3%) | | 0/1 (0.0%) | | | - | | | |  |  |  |
| *TIMP3* | 58 | 3/23 (13.0%) | | 1/12 (8.3%) | 1/7 (14.3%) | | | 0/4 (0.0%) | | 0/1 (0.0%) | | | - | | | |  |  |  |
| *E-cadherins* | 63 | - | | - | 6/16** (37.5%) | | | - | | - | | | - | | | |  |  |  |
| *E-cadherins* | 63 | - | | - | 0/10*** (0.0%) | | | - | | - | | | - | | | |  |  |  |
| *Table S1:* Epigenetic gene regulation (DNA methylation) according to PA histopathological subtype  * Human pituitary cell lines show reduced GH and increased PRL hormone production when IK1 is overexpressed | | | | | | | | | | | | |  | | |  | |  |  |
| ** With fibrous bodies | |  |  |  |  |  |  | |  | |  |  | |  |  | | | |  |
| *** Without fibrous bodies | | |  |  |  |  |  | |  | |  |  | |  |  | | | |  |
