# Supplementary material for: The Role of Epigenetic Modification in Tumorigenesis and Progression of Pituitary Adenomas: A Systematic Review of the Literature
Source: PLoS One. 2013 Dec 18;8(12):e82619. doi: 10.1371/journal.pone.0082619 (PMC3867353; doi:10.1371/journal.pone.0082619)
Supplement: Table S2 — Epigenetic gene regulation (DNA methylation) associated with clinical and tumor characteristics (age, sex, invasiveness). (DOCX) [file pone.0082619.s002.docx]

| **Gene** | | **References** | | | **Overall**  **Proportion Methylated** | | | **Patient Age (years)** | | | | | **Patient Sex** | | | | | | | | **PA invasion status** | | | | |
| --- | --- | --- | --- | --- | --- | --- | --- | --- | --- | --- | --- | --- | --- | --- | --- | --- | --- | --- | --- | --- | --- | --- | --- | --- | --- |
|  |  |  |  |  |  |  |  | **Methylated** | | **Unmethylated** | | | **Male** | | | | **Female** | | | | **Invasive** | | **Noninvasive** | | |
| *Ikaros** | | 8, 52 | | | 16/22 (72.7%) | | | - | | - | | | 10/15 (66.6%) | | | | 5/7 (71.4%) | | | | - | | - | | |
| *GADD45y* | | 57 | | | 19/33 (57.6%) | | | - | | - | | | - | | | | - | | | | 7/14 (50.0%) | | 10/18 (55.6%) | | |
| *CDKN2A* | | 14, 19, 21, 23, 25, 26, 27, 37, 62 | | | 220/393 (56.0%) | | | 50.78 | | 47.07 | | | 61/94 (64.9%) | | | | 60/119 (50.4%) | | | | 53/92 (57.6%) | | 71/140 (50.7%) | | |
| *FGFR2* | | 41 | | | 12/22 (54.5%) | | | - | | - | | | 9/14 (64.3%) | | | | 3/8 (37.5%) | | | | - | | - | | |
| *Caspase 8* | | 58 | | | 19/35 (54.3%) | | | 47.52 | | 43.50 | | | 10/18 (55.6%) | | | | 9/17 (52.9%) | | | | - | | - | | |
| *PTAG* | | 57 | | | 24/71 (33.8%) | | | - | | - | | | - | | | | - | | | | 8/14 (57.1%) | | 10/21 (47.6%) | | |
| *TSP-1* | | 58 | | | 15/35 (42.9%) | | | 47.93 | | 44.00 | | | 8/18 (44.4%) | | | | 7/17 (41.2%) | | | | - | | - | | |
| *RASSF1A* | | 58 | | | 20/52 (38.5%) | | | - | | - | | | 6/25 (24.0%) | | | | 14/25 (56.0%) | | | | 10/23 (43.5%) | | 10/29 (34.5%) | | |
| *Rb1* | | 23, 37, 58, 65 | | | 47/126 (37.3%) | | | 43.90 | | 47.19 | | | 15/53 (28.3%) | | | | 17/52 (32.7%) | | | | 8/15 (53.3%) | | 3/15 (20.0%) | | |
| *p73* | | 58 | | | 14/69 (20.3%) | | | 42.40 | | 47.40 | | | 6/18 (33.3%) | | | | 4/17 (23.5%) | | | | - | | - | | |
| *MGMT* | | 58 | | | 8/33 (24.2%) | | | 49.44 | | 44.38 | | | 7/25 (28.0%) | | | | 1/18 (5.3%) | | | | - | | - | | |
| *E-cadherins* | | 63 | | | 6/26 (23.1%) | | | - | | - | | | - | | | | - | | | | - | | - | | |
| *p14* | | 58 | | | 8/69 (11.6%) | | | 38.02 | | 47.20 | | | 3/18 (16.7%) | | | | 3/17 (17.6%) | | | | - | | - | | |
| *DAP Kinase* | | 13, 16, 58 | | | 9/53 (17.0%) | | | 45.00 | | 45.72 | | | 0/18 (0.0%) | | | | 2/19 (10.5%) | | | | 5/13 (38.5%) | | 2/14 (14.3%) | | |
| *TIMP3* | | 58 | | | 4/35 (11.4%) | | | 47.50 | | 45.45 | | | 2/18 (11.1%) | | | | 2/17 (11.8%) | | | | - | | - | | |
| *p21***** | | 37 | | | 1/34 (2.9%) | | | - | | - | | | - | | | | - | | | | - | | - | | |
| *p27***** | | 37 | | | 0/34 (0.0%) | | | - | | - | | | - | | | | - | | | | - | | - | | |
|  |  | |  | | | |  | |  | |  |  | |  |  | | |  |  |  |  |  |  |  |  |
|  |  | |  | | | |  | |  | |  |  | |  |  | | |  |  |  |  |  |  |  |  |
| *Table S2:* Epigenetic gene regulation (DNA methylation) associated with clinical and tumor characteristics (age, sex, invasiveness).  * Human pituitary cell lines show reduced GH and increased PRL hormone production when IK1 is overexpressed | | | | | | | | | | | | | | | | | | | | | |  | |  |  |
| ** With fibrous bodies,  ***Without fibrous bodies | | | |  | |  | |  | |  | | |  | | |  |  | | |  |  |  | |  |  |
|  | | | | | |  | |  | |  | | |  | | |  |  | | |  |  |  | |  |  |
| **** Validated in mouse models | | | | | |  | |  | |  | | |  | | |  |  | | |  |  |  | |  |  |
